# Institutions for the Insane in Prussia, Austria, and Germany

**Published:** 1854-07

**Authors:** 


### BOOK NOTICES.

*Institutions for the Insane in Prussia, Austria, and Germany.* By PLINY EARLE, M. D., one of the visiting Physicians to the Lunatic Asylum of the City of New York: late Physician to the Bloomingdale Asylum; Fellow of the College of Physicians and Surgeons of New York, and of the New York Academy of Medicine.—New York: S. S. & William Wood, 261 Pearl Street, 1845.

*An Examination of the Practice of Bloodletting in Mental Disorders.*—By PLINY EARLE, M. D., Author of "A Visit to Thirteen Asylums for the Insane in Europe;" "History, Description, and Statistics of the Bloomingdale Asylum for the Insane;" and "Institutions for the Insane in Prussia, Austria, and Germany."—New York: S. S. & William Wood, 261 Pearl Street, 1854.

THE above works are from the pen of one of the most accomplished scholars in the medical profession of our country. The first is a volume of 246 pages, containing a full and very interesting account of the institutions for the Insane in the countries named in the title, together with the prevailing doctrines concerning mental disorders and their treatment. The study and treatment of insanity attracted attention at an early period among the Germans, and it has continued to receive much attention from some of the most learned and eminent men in the German states, up to the present time. Dr. EARLE precedes his account of the Institutions of the several countries named by a very interesting *history* of the *Literature* of mental disorders in the Central states of Europe. We have not time now to give any analysis of this work. To those in our country who are devoting special attention to the subject of insanity, this work of Dr. EARLE is invaluable; to all intelligent readers it would be interesting and profitable, and to the literature of our profession it is a valuable addition.

The second work, the title of which we have given, is of more immediate and practical interest to the whole profession. It is a monograph of 126 pages, containing besides the views of the author, a most laborious and careful collation of the opinions of every writer of note, both in Europe and America, touching the application and value of bloodletting in the treatment of mental disorders. Hence we have here in a small compass, what would otherwise cause the reader an extent of literary research, entirely beyond the reach of most of the members of our profession. And yet it all relates to a practical point of great importance, and one with which every physician is liable to come in contact. Most of the insane are, at first, under the care of the ordinary practitioner of medicine for a longer or shorter period, and it is not unfrequently the case that the all important question of their recovery or permanent derangement, is fixed by the treatment they receive at that time. Every physician, therefore, should be well informed in relation to the actual value of those remedial agencies, which are most generally applied in the early stage of insanity. Among those agencies, bloodletting has ever held a prominent rank, more especially in our country where the writings of Dr. Rush have exerted a wide and lasting influence. Dr. Earle has done the profession a great favor by publishing the little Monograph now before us. And we say, unhesitatingly, that it should be purchased and read by every practitioner in America.

The following are the general conclusions deduced by Dr. Earle from the examination of the subject before us, viz.:

1. Insanity, in any form, is not, of itself, an indication for bloodletting.

2. On the contrary, its existence is, of itself, a contra-indication. Hence, the person who is insane should, other things being equal, be bled less than one who is not insane.

3. The *usual* condition of the brain, in mania, is not that of active inflammation, but of a species of excitement, irritability, or irritation, perhaps more frequently resulting from or accompanied by anaemia, debility, or abnormal preponderance of the nervous over the circulatory functions, than in connexion with plethora and enduring vital power.

4. The excitement, both mental and physical, produced by this irritation, can, in most cases, be permanently subdued, and its radical source removed by other means, more readily than by bleeding.

5. Yet insanity may be coexistent with conditions—such as positive plethora, a tendency to apoplexy or paralysis, and sometimes sthenic congestion or inflammation, which call for the abstraction of blood. Therefore,

6. Venesection in mental disorders should not be absolutely abandoned, although the cases requiring it are very rare.

7. As a general rule, *topical* is preferable to *general* bleedings

8. In many cases where the indication for direct depletion is not urgent, but where bloodletting, particularly if local, might be practiced without injury, it is safer and better to treat by other means; equalizing the circulation and promoting the secretions and excretions.

9. The physical conditions requiring bloodletting more frequently exist in mania than in any other of the ordinary forms of mental alienation.

10. Insanity following parturition, other things being equal, is to be treated by bleeding less frequently than that which has its origin in other causes.

11. If the mental disorder be the direct result of injury to the head, the treatment must be directed to the wound, or its physical effects, not specially to the psychic condition.

12. In many cases where insanity is accompanied by typhus symptoms, and in some where the aspect is that of acute phrenitis, active stimulants alone can save the patient, and direct depletion from the circulation is almost certainly fatal.

